# Assessing time use in long-term institutional care: development, validity and inter-rater reliability of the Groningen Observational instrument for Long-Term Institutional Care (GO-LTIC)

**DOI:** 10.1186/s12912-016-0133-y

**Published:** 2016-02-29

**Authors:** Astrid Tuinman, Mathieu de Greef, Roos Nieweg, Wolter Paans, Petrie Roodbol

**Affiliations:** School of Nursing, Hanze University of Applied Sciences Groningen, Groningen, The Netherlands; Human Movement Sciences, University Medical Center Groningen, University of Groningen, Groningen, The Netherlands; Research Group Nursing Diagnostics, Hanze University of Applied Sciences Groningen, Groningen, The Netherlands; School of Nursing & Health, University Medical Center Groningen, University of Groningen, Groningen, The Netherlands

**Keywords:** Classification, Content validity, Instrument development, Inter-rater reliability, Long-term care, Nursing intervention, Nursing home, Nursing staff, Observation

## Abstract

**Background:**

Limited research has examined what is actually done in the process of care by nursing staff in long-term institutional care. The applied instruments employed different terminologies, and psychometric properties were inadequately described. This study aimed to develop and test an observational instrument to identify and examine the amount of time spent on nursing interventions in long-term institutional care using a standardized language.

**Methods:**

The Groningen Observational instrument for Long-Term Institutional Care (GO-LTIC) is based on the conceptual framework of the Nursing Interventions Classification. Developmental, validation, and reliability stages of the GO-LTIC included: 1) item generation to identify potential setting-specific interventions; 2) examining content validity with a Delphi panel resulting in relevant interventions by calculating the item content validity index; 3) testing feasibility with trained observers observing nursing assistants; and 4) calculating inter-rater reliability using (non) agreement and Cohen’s kappa for the identification of interventions and an intraclass correlation coefficient for the amount of time spent on interventions. Bland-Altman plots were applied to visualize the agreement between observers. A one-sample student *T*-test verified if the difference between observers differed significantly from zero.

**Results:**

The final version of the GO-LTIC comprised 116 nursing interventions categorized into six domains. Substantial to almost perfect kappa’s were found for interventions in the domains basic (0.67–0.92) and complex (0.70–0.94) physiological care. For the domains of behavioral, family, and health system interventions, the kappa’s ranged from fair to almost perfect (0.30–1.00). Intraclass correlation coefficients for the amount of time spent on interventions ranged from fair to excellent for the physiological domains (0.48–0.99) and poor to excellent for the other domains (0.00–1.00). Bland Altman plots indicated that the clinical magnitude of differences in minutes was small. No statistical significant differences between observers (*p* > 0.05) were found.

**Conclusions:**

The GO-LTIC shows good content validity and acceptable inter-rater reliability to examine the amount of time spent on nursing interventions by nursing staff. This may provide managers with valuable information to make decisions about resource allocation, task allocation of nursing staff, and the examination of the costs of nursing services.

## Background

Being confronted with the increasing dependency levels of frail residents and limited budgets, managers of long-term institutional care (LTIC) search for an optimal staff, which means an appropriate number of nursing staff and a mix of staff levels, to enhance or maintain quality of care standards while reducing costs [1].

To gain insight into quality of care, the conceptual model of Donabedian [2] indicates that information regarding structure (e.g., number and type of nurses), process, and outcomes (e.g., pressure ulcers) is needed. The total number of nursing staff in LTIC appears to be associated with better quality of care [3, 4]. However, reviews show mixed results concerning the relationship between the type of nursing staff (e.g., nurses, nursing assistants) and quality of care outcomes [3–5]. Due to the secondary survey data utilized by most studies, the interventions performed by nursing staff in the process of care remained unclear and, therefore, so did their contribution to quality of care outcomes [3–5].

Arling et al. [6] contend that the amount of time spent with a resident has a great impact on quality of care. What is done, how much, by whom, and how, all influences residents’ care [3]. This increases the importance of the deployment of nursing staff in the provision of care [7]. Identifying nurses’ interventions and the amount of time spent on them may clarify their contribution to quality of care and support task allocation to the type of nursing staff according to their specific scope of practice.

According to Donabedian, process is defined as what is actually done in providing and receiving care and this can be assessed by direct observation [2]. Observational studies addressing the process of care in LTIC provide insight into time use of registered nurses [8, 9] and health care aids [8, 10, 11]. Psychometric properties of the applied instruments were either missing or briefly described, and instruments varied in the content and categorization of nursing activities which made it difficult to compare study results.

Instruments based on an internationally known standardized nursing language compared to colloquial terms allow for data aggregation and analysis between settings [12]. A widely used standardized language that defines and categorizes nursing interventions is the Nursing Interventions Classification (NIC). The NIC describes a nursing intervention as any treatment based on the judgment and clinical knowledge of a nurse aiming to increase the recipient’s care outcomes [13]. The NIC provides labels and definitions of interventions and categorization into classes and domains. Per intervention, a list of activities describes the specific nurses’ behaviors or actions [13]. An advantage of the NIC is that it provides estimates of the amount of time to perform the intervention along with the type of nursing staff to deliver the intervention.

Studies have employed the NIC as a framework for identifying interventions for groups of patients in hospitals [14], ambulatory nursing [15], parish nursing [16] and advanced nursing practice [17]. A number of studies used the NIC to describe the amount of time spent on interventions to examine workload [18, 19] or personnel staffing [20]. No studies were found related to LTIC.

The aim of the current study was to develop and test the content validity and inter-rater reliability of an observational instrument using the NIC as a conceptual framework in order to identify and examine the amount of time spent on nursing interventions in LTIC.

## Methods

Several stages have been completed to develop and test the observational instrument based on recommendations by Streiner et al. [21, 22]. The stages were: 1) item generation; 2) examining content validity; 3) testing feasibility; and 4) inter-rater reliability assessment.

### Population, setting and sampling

The population was nursing staff working in LTIC. A purposive sample was performed to provide for a diversity of facilities, units, and personnel. In total, four nursing homes, two care centers (combined residential care and nursing home), and three residential care homes in the north of the Netherlands consented to participation. The recruitment of nursing staff working in different types of units (somatic, psycho-geriatric, and residential care) was performed in cooperation with facility managers. The inclusion criterion was at least one year of working experience in LTIC.

### Data collection

#### Stage 1 Item generation

The NIC described 542 interventions classified into 30 classes and seven domains [23]. Potential study setting-specific nursing interventions were identified by observing nursing staff during day shifts. Bachelor nursing students (5) in their final year of education and the principal investigator (AT) (further referred to as research team), all with expertise in long-term care (average working experience of two years) and knowledge of the NIC, conducted the observations without a predefined list of activities. Afterwards, the observed care activities were linked to NIC interventions, which resulted in an initial inventory of interventions that was presented to a Delphi panel.

#### Stage 2 Content validity

A two-round postal Delphi survey was conducted to obtain consensus on the relevance of the initial inventory. Nine experts including five registered nurses and four nursing assistants of participating facilities agreed to contribute. Experience with the NIC was not a prerequisite. The survey comprised concept labels and definitions per NIC intervention. In the first Delphi round, experts were asked to rate the relevance of each intervention by the frequency of occurrence in their facility on a 5-point Likert scale (1 = never; 2 = rarely, less than one time per week; 3 = sometimes, more than one time per week, but less than every day; 4 = often, one time every day; and 5 = very often, more than once per day). An additional column was included for comments.

The second Delphi round comprised interventions on which no consensus was obtained to either include or exclude in the observational instrument. This time, experts were asked to rate an intervention as: 1 = “relevant, could have occurred in the last three weeks”, or 2 = “not relevant”.

#### Stage 3 Feasibility

The feasibility test was performed to support the Delphi results and to test the data collection method to be used (structured continuous observations) [24]. As a component of the data collection method, five observers (nursing students of the research team) who had gained basic knowledge of the NIC through their professional education were trained during three two-hour sessions. They individually mapped the interventions that were performed by nurses in video fragments to NIC interventions. The mapping procedure implied that an observed intervention, comprising specific nurses’ activities, was linked to the most accurate NIC intervention by comparison of relevant intervention labels and definitions. Discrepancies between observers were discussed until consensus was reached on which NIC intervention was most appropriate, and a log of these decisions was kept. An interventions’ duration was recorded by writing start and end times using a stopwatch. The mapping procedure was subsequently tested in a residential care home and nursing home where two observers simultaneously observed one nursing assistant continuously during a day shift.

#### Stage 4 Inter-rater reliability

Continuous observations of nursing staff took place in two care centers, two residential care homes, and a nursing home. Different types of nursing staff were observed during day shifts in different types of units. Observations took place with four (out of five) paired observers whereby the combination alternated. Observers linked their observations independently to NIC interventions according to the mapping procedure.

### Statistical analyses

#### Stage 2 Examining content validity

Descriptive statistics were used to present the characteristics of the Delphi experts. Based on the ratings of the experts, the content validity was computed on the item level for each NIC intervention with the item content validity index (I-CVI) and on the scale level for NIC domains with the scale content validity index (S-CVI) [24] in Microsoft Excel® 2010 (Microsoft Corp., Redmond, WA). The I-CVI was computed as the number of experts rating a 3, 4, or 5 divided by the total number of experts which is the proportion of agreement per intervention [24]. The S-CVI was obtained by averaging the proportion of items that were rated as relevant across the experts and divided by the number of items, the S-CVI/Ave. An I-CVI of 0.80 was considered acceptable [24] whereby the intervention was included in the observational instrument. An S-CVI/Ave of 0.90 was considered acceptable [24].

#### Stage 4 Inter-rater reliability assessment

The interventions’ duration in minutes was entered into IBM SPSS Statistics 19 (Armonk, NY: IBM Corp). Interventions were categorized into the NIC domains. Inter-rater reliability was computed for each observer pair per domain. Inter-rater agreement for the identification of interventions, meaning the extent to which observers mapped observed activities to the same NIC interventions, was calculated by (non) agreement percentages with 95 % confidence intervals (CI). In order to do so, the time recordings of the ratio scale were dichotomized per intervention (0 = time noted, 1 = no time noted). The (non) agreement was calculated to determine whether observers agreed when care did or did not occur [25]. So as not to overestimate the level of agreement, a Cohen’s kappa (unweighted) with a 95 % CI was also calculated. A kappa (K) value of 0–0.20 was considered as slight agreement; 0.21–0.40 as fair; 0.41–0.60 as moderate; 0.61–0.80 as substantial; and 0.81–1 as an almost perfect agreement [26].

To verify the level of inter-rater reliability of time spent on interventions, an intra-class correlation coefficient (ICC) was computed using a two-way random effects model with absolute agreement. Single measures with a 95 % CI are reported. Values less than 0.40 were considered poor; between 0.40 and 0.59 as fair; 0.60 and 0.74 as good; and between 0.75 and 1.0 as excellent [27].

Bland-Altman plots were used to visualize and quantify agreement between all paired observations per domain. Means and 95 % limits of agreement were calculated and provided visual judgement of how well observers agreed on the amount of time spent on a domain. A smaller range between the upper and lower limits indicates a better agreement. A range of agreement is defined as a mean bias ±1.96 standard deviation (SD) [28, 29]. A one-sample student *T*-test was performed in order to examine if the difference between observers differed significantly from zero, indicating fixed bias. The statistical significance level was set at *p* < 0.05.

### Ethical considerations

This study was conducted in accordance with the guidelines of Good Clinical Practice [30] which principles have their origin in the Declaration of Helsinki [31]. Approval was obtained from the Medical Ethics Review Board of the University Medical Center Groningen, The Netherlands. Informed consent was obtained from the residents or their legal representatives to allow observers entrance to residents’ rooms. Facility managers did not allow that the two observers entered psycho-geriatric units at the same time as this was considered too disruptive for these residents with cognitive impairments.

## Results

**Fig. 1:**
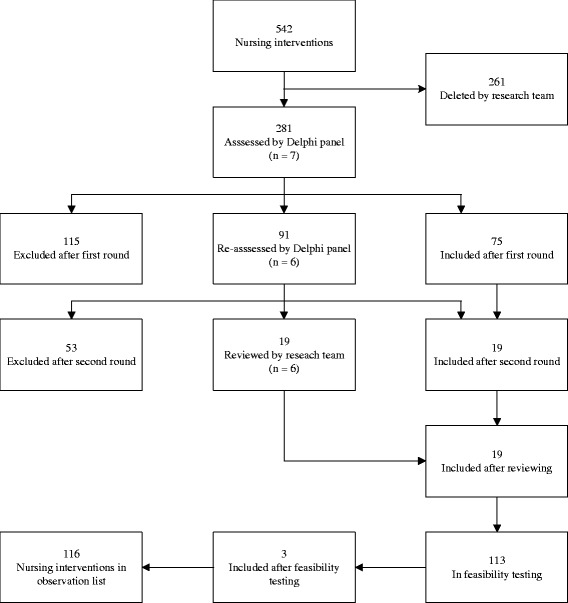
Flowchart of instrument development

The results follow the chronological order in which the four stages occurred. A flowchart of the instruments’ development is provided (Fig. 1).

The initial observations of nurses’ activities were linked to 281 (out of 542) potentially setting-specific NIC interventions resulting in an inventory that was forwarded to the nine experts of the Delphi panel in the first round.

Seven experts responded in the first round. Their median age was 32 (interquartile range [IQR] 25) and working experience five years (IQR 17.5) (Table 1). The experts concurred on 75 interventions that frequently occur in LTIC (I-CVI ≥ 0.86) (Fig. 1). Their written comments suggested the inclusion of another 91 interventions with an I-CVI of 0.57 or 0.71. These 91 interventions were again sent to the seven experts in the second round. Then, six experts with a median age of 27 (IQR 26) years and a working experience of four years (IQR 15.6) (Table 1) responded. Following this, nineteen interventions with an I-CVI ≥ 0.83 were added to the observational instrument (Fig. 1). Subsequently, interventions with an I-CVI of 0.50 and 0.67 (19) were critically reviewed by the research team. Considering their individual experience in long-term care, the research team considered these interventions as relevant (Fig. 1). With this inclusion, the observational instrument comprised 113 interventions (Fig. 1) in 24 classes and six domains (Table 2). The S-CVI/Ave of domains ranged from 0.79 to 0.93. An overview of included NIC domains and classes with examples of interventions is provided in Table 2.

**Table 1:** Expert characteristics and response to Delphi rounds

Expert	1	2	3	4	5	6	7
Gender	female	female	male	female	female	female	female
Age	46	32	41	21	22	21	50
Educational level^a^	RN	NA	NA	RN	RN	RN	NA
Working experience	5	11	20	2,5	3	1	38
Type LTIC^b^	CC	NH	CC	NH	RC	NH	RC
Response round 1	X	X	X	X	X	X	X
Response round 2	X	X	-	X	X	X	X

^a^ *RN* registered nurse, *NA* nursing assistant

^b^ *LTIC* long-term institutional care, *CC* care centre with residential care, somatic- and psycho-geriatric units, *NH* nursing home with somatic and psycho-geriatric units, *RC* residential care home

**Table 2:** Included NIC^a^ domains and classes with two examples of interventions per class

Domains	Definition domain	Classes	Examples of interventions (NIC code)
Physiological: basic	Care that supports physical functioning	Self-care facilitation, elimination management, immobility management, nutrition support, activity and exercise management, physical comfort promotion.	Self-care assistance (1800), bathing (1610), tube care: urinary (1876), urinary incontinence care (0610), positioning (0840), transfer (0970), feeding (1050), nutritional monitoring (1160), body mechanics promotion (0140), energy management (0180), pain management (1400), environmental management: comfort (6482).
Physiological: complex	Care that supports homeostatic regulation	Electrolyte and acid–base management, drug management, skin/wound management, neurologic management, respiratory management, thermoregulation, tissue perfusion management.	Hyper- and hypoglycemia management (2120/2130), medication administration (2300), medication management (2380), pressure ulcer prevention (3540), skin surveillance (3590), unilateral neglect management (2760), aspiration precautions (3200), asthma management (3210), temperature regulation (3900), fever treatment (3740), fluid management (4120), circulatory care: venous insufficiency (4066).
Behavioral	Care that supports psychosocial functioning and facilitates life style changes	Behavior therapy, cognitive therapy, communication enhancement, coping assistance, patient education, psychological comfort promotion.	Activity therapy (4310), behavior management (4350), memory training (4760), reality orientation (4820), active listening (4920), socialization enhancement (5100), security enhancement (5380), activity therapy (4310), socialization enhancement (5100), support system enhancement (5440), emotional support (5270), teaching: prescribed medication (5616), teaching: disease process (5602), anxiety reduction (5820), calming technique (5880).
Safety	Care that supports protection against harm	Risk management	Fall prevention (6490), elopement precautions (6470).
Family^b^	Care that supports the family	Lifespan care	Home maintenance assistance (7180)
Health System	Care that supports effective use of the health care delivery system	Health system mediation, health system management, information management.	Case management (7320), visitation facilitation (7560), preceptor: student (7726), delegation (7650), shift report (8140), documentation (7920).

^a^ *NIC* Nursing Interventions Classification

^b^Only comprising the intervention home maintenance assistance

The feasibility test revealed three additional interventions that frequently occurred in practice: spiritual support (praying), circulatory care: venous insufficiency (e.g., compression therapy), and airway management (e.g., teach usage of prescribed inhalers). This resulted in a final observational instrument of 116 interventions – the GO-LTIC (Groningen Observational instrument for Long-Term Institutional Care).

Concerning the mapping procedure, it appeared that the definition and label of NIC interventions was not always clear enough to assign an observation to, for instance, when to classify an intervention as ‘dressing’ or ‘self-care assistance’. After a consensus discussion with all of the observers it was decided which was the most accurate fit. Consensus discussions continued during the stage of inter-rater reliability testing if necessary. The usability of the GO-LTIC was improved by organizing NIC classes on frequency of occurrence. It was decided that time recordings were rounded to 30 s.

Regarding inter-rater reliability, four nursing assistants, two primary caregivers (nursing assistants with additional training in coordinating care), and one registered nurse were observed during seven day shifts. They performed interventions on 108 residents in four somatic units (*n* = 44) and three residential care units (*n* = 62). Two residents’ units were unknown. Residents’ average age was 87.1 years; they were primarily female (*n* = 81). From the 116 interventions, 55 were identified by observers, and the amount of time was registered (Table 3). Unobserved interventions mainly concerned the safety and behavioral domains.

**Table 3:** Overview of identified interventions and number of observations

	Interventions in domain	Interventions identified (% of domain)		Number of observations (O1 and O2^a^)
Domain Physiological: basic	47	25	(53)	529
Domain Physiological: complex	20	12	(60)	232
Domain Behavioral	28	8	(29)	72
Domain Safety	6	1	(17)	6
Domain Family	1	1	(100)	180
Domain Health System	14	8	(57)	336
Total domains	116	55	(47)	1355

^a^ *O1* observer 1, *O2* observer

The inter-rater agreement for the identification of interventions yielded from 0.93 to 1.00 except for interventions in the family domain (Table 4). When corrected for chance, substantial to almost perfect agreement was perceived within the domains of basic physiological care (K = 0.67, CI: 0.54–0.81 to K = 0.92, CI: 0.84–0.99) and complex physiological care (K = 0.70, CI: 0.42–0.99 to K = 0.94, CI: 0.82–1.00) (Table 3). Values were fair to almost perfect agreement in the behavioral domain (K = 0.40, CI: 0.00–1.00 to K = 1.00, CI: 1.00), family domain (K = 0.40, CI: 0.12–0.77 to K = 1.00, CI: 0.74–1.00), and health system domain (K = 0.30, CI: 0.00–0.77 to K = 0.76, CI: 0.62–0.90). Interventions in the safety domain were often not identified, resulting in few time recordings, therefore kappa could not be calculated.

**Table 4:** Point estimates of inter-rater reliability tests per NIC domain

Domain labels (number of observations^a^)	Number of residents	Occurrence (CI)^b^	Non-occurrence (CI)^b^	Cohen’s Kappa (CI)^b^	ICC Single (CI)^b^
Physiological: basic, 47 interventions					
Observers 3 & 4 (47*11 = 517)	11	0.97 (0.96–0.98)	0.97 (0.95–0.98)	0.78 (0.67–0.89)	0.64 (0.14–0.89)
Observers 2 & 4 (470)	10	0.99 (0.98–1.00)	0.99 (0.97–1.00)	0.92 (0.84–0.99)	0.94 (0.54–0.99)
Observers 3 & 1 (517)	11	0.96 (0.94–0.97)	0.96 (0.93–0.97)	0.67 (0.54–0.81)	0.97 (0.88–0.99)
Observers 1 & 2 (846)	18	0.99 (0.99–1.00)	0.99 (0.98–1.00)	0.83 (0.69–0.97)	0.87 (0.69–0.95)
Observers 1 & 2 (658)	14	0.98 (0.97–0.99)	0.98 (0.96–0.99)	0.79 (0.69–0.90)	0.95 (0.83–0.99)
Observers 3 & 4 (987)	21	0.99 (0.98–1.00)	0.99 (0.98–1.00)	0.82 (0.70–0.94)	0.99 (0.99–1.00)
Observers 3 & 4 (1081)	23	0.99 (0.99–1.00)	0.99 (0.99–1.00)	0.76 (0.60–0.93)	0.82 (0.63–0.92)
Physiological: complex, 20 interventions					
Observers 3 & 4 (20*11 = 220)	11	0.99 (0.97–1.00)	0.99 (0.97–1.00)	0.90 (0.77–1.00)	0.81 (0.33–0.95)
Observers 2 & 4 (200)	10	0.99 (0.97–1.00)	0.99 (0.97–1.00)	0.94 (0.82–1.00)	0.67 (0.16–0.91)
Observers 3 & 1 (220)	11	0.98 (0.95–0.99)	0.98 (0.95–0.99)	0.70 (0.42–0.99)	0.58 (0.05–0.87)
Observers 1 & 2 (360)	18	0.98 (0.97–0.99)	0.98 (0.97–0.99)	0.81 (0.64–0.98)	0.48 (0.07–0.76)
Observers 1 & 2 (280)	14	0.98 (0.96–0.99)	0.98 (0.96–0.99)	0.70 (0.43–0.96)	0.93 (0.81–0.98)
Observers 3 & 4 (420)	21	0.99 (0.98–1.00)	0.99 (0.98–1.00)	0.88 (0.74–1.00)	0.59 (0.23–0.81)
Observers 3 & 4 (460)	23	0.99 (0.98–1.00)	0.99 (0.98–1.00)	0.89 (0.77–1.00)	0.72 (0.44–0.87)
Behavioral, 28 interventions					
Observers 3 & 4 (28*11 = 308)	11	0.99 (0.98–1.00)	0.99 (0.98–1.00)	0.50 (0.00–1.00)	0.99 (0.95–1.00)
Observers 2 & 4 (280)	10	0.99 (0.97–1.00)	0.99 (0.97–1.00)	0.40 (0.00–1.00)	0.47 (−0.09–0.83)
Observers 3 & 1 (308)	11	1.00 (0.98–1.00)	1.00 (0.98–1.00)	0.86 (0.57–1.00)	0.43 (−0.25–0.81)
Observers 1 & 2 (504)	18	0.99 (0.98–1.00)	0.99 (0.98–1.00)	0.78 (0.58–0.97)	0.89 (0.73–0.96)
Observers 1 & 2 (392)	14	0.99 (0.97–1.00)	0.99 (0.97–1.00)	0.75 (0.50–0.99)	0.93 (0.80–0.98)
Observers 3 & 4 (588)	21	1.00 (0.99–1.00)	1.00 (0.99–1.00)	0.75 (0.40–1.00)	0.21 (−0.22–0.58)
Observers 3 & 4 (644)	23	1.00 (0.99–1.00)	1.00 (0.99–1.00)	1.00 (1.00)	0.00 (−0.40–0.40)
Safety, 6 interventions					
Observers 3 & 4 (6*11 = 66)	11	0.98 (0.92–1.00)	0.98 (0.92–1.00)	0.66 (0.00–1.00)	0.29 (−0.33–0.74)
Observers 2 & 4 (60)	10	1.00 (0.94–1.00)	1.00 (0.94–1.00)	—^c^	0.00 (−0.60–0.60)
Observers 3 & 1 (66)	11	1.00 (0.95–1.00)	1.00 (0.95–1.00)	—	—
Observers 1 & 2 (108)	18	1.00 (0.97–1.00)	1.00 (0.97–1.00)	—	—
Observers 1 & 2 (84)	14	0.99 (0.94–1.00)	0.99 (0.94–1.00)	0.00 (0.00–1.00)	—
Observers 3 & 4 (126)	21	1.00 (0.97–1.00)	1.00 (0.97–1.00)	—	—
Observers 3 & 4 (138)	23	0.99 (0.96–1.00)	0.99 (0.96–1.00)	0.00 (0.00–1.00)	0.00 (−0.40–0.40)
Family, 1 intervention					
Observers 3 & 4 (1*11 = 11)	11	0.91 (0.62–0.98)	0.83 (0.44–0.97)	0.82 (0.48–1.00)	0.43 (−0.14–0.80)
Observers 2 & 4 (10)	10	0.70 (0.40–0.89)	0.40 (0.12–0.77)	0.40 (0.00–0.97)	0.41 (−0.13–0.80)
Observers 3 & 1 (11)	11	0.82 (0.52–0.95)	0.67 (0.30–0.90)	0.65 (0.20–1.00)	0.80 (0.38–0.94)
Observers 1 & 2 (18)	18	1.00 (0.82–1.00)	1.00 (0.77–1.00)	1.00 (1.00)	0.99 (0.97–1.00)
Observers 1 & 2 (14)	14	0.93 (0.69–0.99)	0.80 (0.38–0.96)	0.84 (0.53–1.00)	0.94 (0.82–0.98)
Observers 3 & 4 (21)	21	1.00 (0.85–1.00)	1.00 (0.74–1.00)	1.00 (1.00)	0.24 (−0.18–0.60)
Observers 3 & 4 (23)	23	0.87 (0.68–0.96)	0.84 (0.62–0.95)	0.64 (0.27–1.00)	1.00 (−)
Health System, 14 interventions					
Observers 3 & 4 (14*11 = 154)	11	0.99 (0.95–1.00)	0.99 (0.95–1.00)	0.66 (0.19–1.00)	0.38 (−0.20–0.78)
Observers 2 & 4 (140)	10	0.94 (0.89–0.97)	0.94 (0.89–0.97)	0.30 (0.00–0.77)	0.96 (0.85–0.99)
Observers 3 & 1 (154)	11	0.94 (0.89–0.97)	0.94 (0.89–0.97)	0.65 (0.20–1.00)	0.12 (−0.48–0.65)
Observers 1 & 2 (252)	18	0.99 (0.97–1.00)	0.99 (0.97–1.00)	0.57 (0.08–1.00)	0.03 (−0.38–0.46)
Observers 1 & 2 (196)	14	0.93 (0.89–0.96)	0.93 (0.88–0.96)	0.63 (0.44–0.82)	0.84 (0.57–0.95)
Observers 3 & 4 (294)	21	0.99 (0.97–1.00)	0.99 (0.97–1.00)	0.40 (0.00–1.00)	0.73 (0.44–0.88)
Observers 3 & 4 (322)	23	0.97 (0.94–0.98)	0.96 (0.94–0.98)	0.76 (0.62–0.90)	0.64 (0.33–0.83)

*NH* nursing home, *CC* care centre, combining psycho-geriatric, somatic, and residential care units, *RC* residential care home, *NA* nursing assistant, *PCG* primary caregiver, NA with additional training, *RN* registered nurse

^a^Including occurrence + non-occurrence + disagree

^b^ *CI*95 % confidence interval

^c^— = not possible to calculate due to too many zero’s caused by a limited number of observations

Good to excellent inter-rater reliability for the time spent on interventions was found for the domain of basic physiological care (ICC = 0.64, CI: 0.14–0.89 to ICC = 0.99, CI: 0.99–1.00) and fair to excellent for the domain complex physiological care (ICC = 0.48, CI: 0.07–0.76 to ICC = 0.93, CI: 0.81–0.98). Poor to excellent values were found for the domains behavioral (ICC = 0.00, CI: −0.40–0.40 to ICC = 0.99, CI: 0.95–1.00), safety (ICC = 0.00, CI: −0.40–0.40 to ICC = 0.29, CI: −0.33–0.74), family (ICC = 0.24, CI: −0.18–0.60 to ICC = 1.00, CI: −) and health system (ICC = 0.03, CI: −0.38–0.46 to IC = 0.96, CI: 0.85–0.99).

**Fig. 2:**
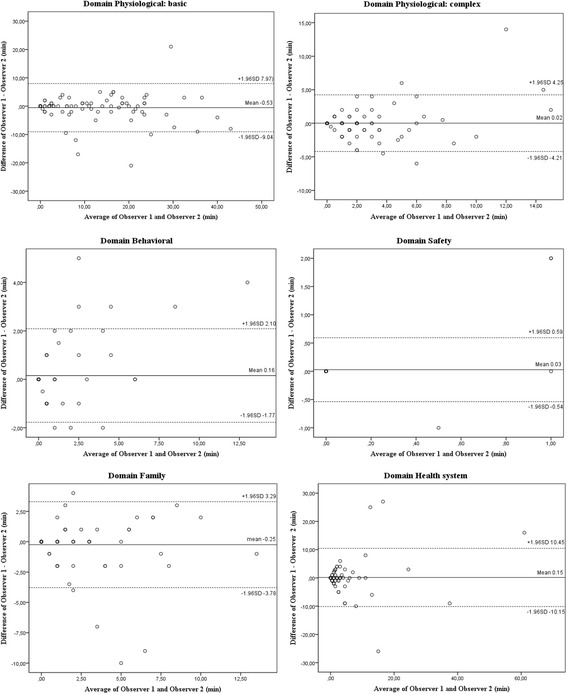
Bland-Altman plots with mean differences (solid lines) and 95 % confidence intervals (dashed lines) in minutes

Bland-Altman plots illustrated differences between observers’ paired observations. The mean differences in domains were: physiological basic 0.53 min (SD 4.34), physiological complex 0.02 min (SD 2.16), behavioral 0.16 (SD 0.99), safety 0.03 (SD 0.29), family −0.25 (SD 1.81), and health system 0.15 min (SD 5.25) (Fig. 2). The one-sample student *T*-test indicated no significant differences between observers (*p* > 0.05).

## Discussion

This study shows that the GO-LTIC has good content validity and acceptable inter-rater reliability to identify nursing interventions and the amount of time spent on these in LTIC. Based on the conceptual framework of the NIC, the instrument comprises 116 interventions categorized into 24 classes and six domains.

Though the content validity of the GO-LTIC was good (I-CVI ≥ 0.80) for most interventions (*n* = 94), a limited number of interventions (*n* = 19) showed a value lower than the cut-off point (0.80). A low I-CVI can mean that experts were not sufficiently proficient [32]. Only working experience was an inclusion criterion. The experts’ identification of interventions may have been complicated since the terms employed in a standardized nursing language such as the NIC lack complete alignment between terms that nurses use during their daily practice [33].

With the exception of interventions in the family domain, reliability assessment concerning the identification of interventions yielded, inter-rater agreements from 0.93 to 1.00, which is in concordance with observational LTIC studies of Dellefield et al. [9] (0.82–0.85) and Munysia et al. [34] (0.90). In order to claim adequate inter-rater reliability, agreement should be 0.90 [35]. When corrected for chance, inter-rater reliability varied between ‘almost perfect’ for the physiological domains (K = 0.67–0.94) and from ‘slight agreement’ to ‘almost perfect’ for the other domains (K = 0.30–1.00). This is lower than a study of Cardona et al. [36] who found a Cohen’s kappa of 0.88. An explanation may be that Cardona et al. [36] used work sampling as a data collection technique while this study conducted structured continuous observations which are labor-intensive [37], therefore, data collector fatigue may have resulted in less accurate recordings. However, in time studies, this technique should be considered as it is more accurate especially when results can affect policy decisions concerning, for example, task allocation [37]. In this study, no data were obtained in psycho-geriatric units which may have resulted in fewer observations, especially in the safety and behavioral domains (e.g., elopement precautions, behavior management). Because the number of observations (= prevalence) influences Cohen’s kappa [38], this may explain the lower values in these domains.

In addition, the observational instrument of Cardona et al. [36] comprised 24 interventions specifically for the use in a locked unit where residents exhibited disruptive behavior. The GO-LTIC comprises 116 interventions for the purpose of examining the time use of nursing staff in different types of units. Ferketich [39] contends that instruments should have a minimal length and represent a specific population and purpose while achieving acceptable support for their reliability and validity. The GO-LTIC showed good content validity and acceptable inter-rater reliability, therefore, it was decided not to exclude any interventions. Furthermore, it has been argued that a greater set of activities in time studies is feasible when data are collected by continuous observations because one observer will observe only one subject [37].

The inter-rater reliability for the amount of time spent on interventions varied, and ICC’s ranged from fair to excellent for the physiological domains (0.48–0.99) and poor to excellent for the other domains (0.00–1.00). Bland Altman plots indicated that the clinical magnitude of most differences in minutes was small. Only the standard deviation of the domains physiological basic and health system exceeded the a priori set acceptable mean bias of 1.96 SD. In addition, a one-sample student *T*-test showed no statistical significant differences between observers.

Structured observations require trained observers with knowledge of the phenomena under investigation and pretesting of instruments in addition to a category system for classifying [24]. In this study, observers with a nursing background were recruited and trained to map activities performed by nursing staff to the most accurate NIC intervention. This, followed by the feasibility test, contributed to the reliability. An advantage of the GO-LTIC is that it is based on a standardized language whereby the work of staff is uniformly represented. This may increase the comparability of studies and, furthermore, could promote benchmarking of LTIC facilities at local, regional, national, and international levels [33]. The instrument shows good content validity and acceptable reliability in the Dutch LTIC context. As instruments are continuously being used in different circumstances and with other groups of people, reliability and validity are never ending processes [22].

## Conclusion

This study describes the potential of the GO-LTIC for examining what interventions nursing staff spend their time on during the process of care. The instrument demonstrates good content validity in the Dutch LTIC context. When the observations are conducted by adequately trained observers with a nursing background, the instrument shows acceptable inter-rater reliability. The value of the GO-LTIC is that it allows for the identification of nursing interventions that are performed for a specific population which could also increase the visibility of nursing staffs’ contribution to quality of care outcomes. Furthermore, if it is known who is doing what and the time involved with this, the GO-LTIC has the potential to enable managers’ decisions regarding task allocation of nursing staff according to their specific scope of practice, resource allocation, and the examination of the costs of services. Furthermore, by using a standardized nursing language, the GO-LTIC may be valuable to the analysis across settings and promote benchmarking of LTIC facilities at local, regional, national, and international levels.
